# On the Rational Drug Design for Hypertension through NMR Spectroscopy

**DOI:** 10.3390/molecules26010012

**Published:** 2020-12-22

**Authors:** Eleni Chontzopoulou, Andreas G. Tzakos, Thomas Mavromoustakos

**Affiliations:** 1 Department of Chemistry, National and Kapodistrian University of Athens, 15784 Athens, Greece; elenichontzo@chem.uoa.gr; 2 Department of Chemistry, Section of Organic Chemistry and Biochemistry, University of Ioannina, 45110 Ioannina, Greece; atzakos@uoi.gr

**Keywords:** NMR spectroscopy, AT1R antagonists, lipid bilayers, conformational properties, drug formulation, polymers, micelles

## Abstract

Antagonists of the AT1receptor (AT1R) are beneficial molecules that can prevent the peptide hormone angiotensin II from binding and activating the specific receptor causing hypertension in pathological states. This review article summarizes the multifaced applications of solid and liquid state high resolution nuclear magnetic resonance (NMR) spectroscopy in antihypertensive commercial drugs that act as AT1R antagonists. The 3D architecture of these compounds is explored through 2D NOESY spectroscopy and their interactions with micelles and lipid bilayers are described using solid state ^13^CP/MAS, ^31^P and ^2^H static solid state NMR spectroscopy. Due to their hydrophobic character, AT1R antagonists do not exert their optimum profile on the AT1R. Therefore, various vehicles are explored so as to effectively deliver these molecules to the site of action and to enhance their pharmaceutical efficacy. Cyclodextrins and polymers comprise successful examples of effective drug delivery vehicles, widely used for the delivery of hydrophobic drugs to the active site of the receptor. High resolution NMR spectroscopy provides valuable information on the physical-chemical forces that govern these drug:vehicle interactions, knowledge required to get a deeper understanding on the stability of the formed complexes and therefore the appropriateness and usefulness of the drug delivery system. In addition, it provides valuable information on the rational design towards the synthesis of more stable and efficient drug formulations.

## 1. Introduction

Angiotensin II Type 1 receptor (AT1R) antagonists are a class of molecules that antagonize the detrimental action of the octapeptide hormone Angiotensin II (AII) that acts on the *G protein*-coupled *receptor* (GPCR) AT1 and induces hypertension [1,2]. The first drug that was developed, acting as an AT1R antagonist was losartan. After that, the class of molecules targeting AT1R is named SARTANs. Sartans are well known molecules, widely prescribed for the treatment of hypertension and a variety of different diseases [3,4,5,6]. The molecular target of AT1R antagonists has been recently established by the crystallization of these molecules in the receptor’s active site. However, their mechanism of action still remains unknown [7,8]. These molecules could exert their action either directly on the AT1R or indirectly, through a two-step mechanism where membrane bilayers play a significant role in molecule’s binding to the receptor. According to the aforementioned mechanism of action, sartans are initially embedded in the lipid bilayers and then they are diffused in the active site of the receptor [9,10,11,12,13,14].

Our laboratory is actively involved in the comprehensive understanding of the molecular and mechanistic basis of the action of different sartans through the application of a battery of biophysical techniques. Existing evidence is still not unequivocal as far as differentiating between the two mechanisms of action [15,16]. Discovering the exact mechanism that the molecules use to approach their receptors could be an extremely important tool in the drug design process. The design of novel molecules should take into account, not only the binding affinity of the drugs to their receptors, but also their capacity to penetrate lipid bilayers or “survive” in the aquatic environment [17]. Moreover, many obstacles like the accumulation of drug molecules into the cellular membrane [18] could be surpassed by observing the mechanism each drug utilizes to reach its receptor’s active site. In order to unveil the molecular basis of drug’s action and improve its efficiency, exploring the role of membranes in drugs’ binding could provide insightful information for the stereoelectronical requirements of novel drugs. Scientific groups worldwide seek for identifying the potent role of the cellular membrane in the drug binding to a variety of different receptors [9]. According to Paul D. Dobson et al., the biophysical forces that govern the interaction of drugs with lipid membranes are similar to those involved in their interaction with proteins, thus it would be a mistake to ignore them [19]. An important tool to investigate such interactions is NMR spectroscopy. Makriyannis et al. propose a combination of ^2^H solid state (ss) NMR and small-angle X-ray diffraction in order to obtain the atomic details of the binding between lipophilic cannabinergic ligands and lipid bilayers [10,20]. Drug design targeting the AT1R (GPC receptor [21,22]) is one of the most popular cases where the role of the lipid bilayers has been investigated. Important information should be revealed from such studies so as to lead the drug design process towards more pharmacologically active compounds [23]. Herein, we will outline different aspects in rational drug design through the use of high-resolution nuclear magnetic resonance (NMR) spectroscopy in the liquid and the solid state.

## 2. Conformational Properties, Drug Action and Stability of AT1R Antagonists in Solvents

An important aspect to design novel therapeutic drugs against hypertension is the investigation of the endogenous ligand’s binding to the receptor and the extensive study of its interactions with critical residues on the receptor’s cavity. NMR is a powerful tool to study atomic interactions, hence it could provide very fruitful information concerning the important interactions of the binding. In 2002, D’Amelio et al. conducted 2D NOESY experiments in order to monitor angiotensin’s binding to a specific region of AT1R, the fragment peptide 300–320 (fCT300–320) of the rat angiotensin II receptor AT1a. The NOE effects observed for angiotensin II in complex with AT1R’s fragment were quite different in comparison with the NOEs of angiontensin alone in solution. Moreover, D’ Amelio’s research group indicated that the aromatic residues of angiontensin molecule play a key role in the formation of AT1R-angiontesin II complex. These findings observed by 2D NOESY NMR were further investigated by the chemical shift index (CSI) method and circular dichroism (CD) experiments, revealing the potent conformational helical changes of the receptor, after ligand’s binding [24].

**Figure 1:**
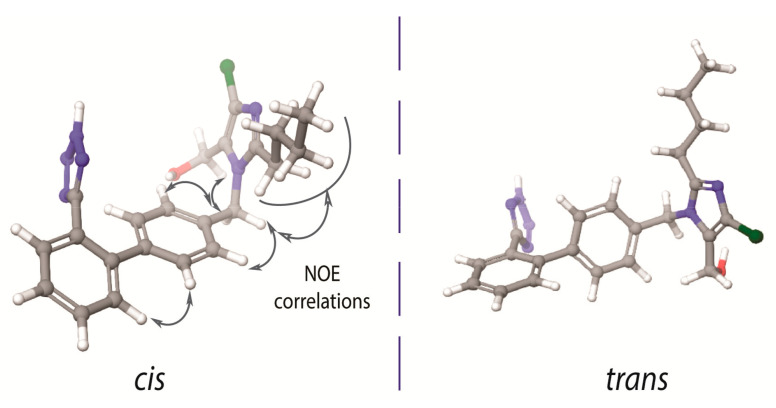
*Cis* and *trans* conformers of losartan. The nuclear Overhauser effect (NOE) between the two aromatic rings depicts absence of their orthogonality. (**left**) The imidazole ring and the tetrazole ring are placed in the same side of the molecule, with respect to the biphenyl ring spacer. (**right**) The imidazole ring and the tetrazole ring are placed in the opposite side of the molecule, with respect to the biphenyl ring spacer.

In 1999 our scientific group initiated a research project concerning the conformational properties of losartan and its peptide antagonist, sarmesin, in order to unveil potent common pharmacophore features of the two AT1R antagonists. Indeed, superimposition of the two molecules revealed that losartan indicates some common structural features with sarmesin (i.e., C-terminal amino acids Ile^5^-His^6^-Pro^7^-Phe^8^-COO- of sarmesin and the corresponding alkyl chain, hydroxyl methyl imidazole, spacer phenyl ring and phenyl tetrazole groups of losartan). This finding explained the rational design of losartan which is based on mimicking the C-terminal of AII [17]. The most important finding in this work was that losartan exists in two possible bioactive enantiomeric conformers as illustrated in Figure 1. However, the biologically important conformation between these two has not yet been identified. This identification is a hard task since these are enantiomeric conformers that cannot be individually evaluated in vitro.

There are some important spatial characteristic features in losartan, that have been revealed by 2D NOESY NMR spectroscopy. As it is well known 2D NOESY provides the spatial correlations between protons and it is an essential experiment in order to study the conformational properties of any molecule in a specific environment. The important nuclear Overhauser effect (NOE) spatial correlations are reported for the one of the two possible bioactive conformers illustrated in Figure 1. The spatial vicinity of the n-butyl chain and the spacer phenyl ring indicates that the hydrophobic chain is oriented above the spacer phenyl ring in order to maximize the hydrophobic interactions of the molecule. NOEs could not be differentiated neither when the imidazole and tetrazole rings are placed in the opposite direction (defined as *trans*) nor when placed in the same direction (defined as *cis*) with respect to the phenyl ring spacer (Figure 1). Although theoretical calculations indicate the favored formation of the *trans* conformer, it has not been yet established if this conformation is adopted into a lipid bilayer or in the receptor’s active site. In order to resolve this important issue our laboratory performs Molecular Dynamics (MD) studies so as to establish the structural preference for the two sartans’ conformers both in the lipid bilayers and in the active site of the receptor. According to a recent publication by our research group, candesartan, another sartan molecule, shows distinct preference to adopt a *cis* conformation in lipid bilayers’ environment [16]. Furthermore, another crucial insight into losartan’s bioactive conformation is an observed NOE between the two spacer phenyl rings which prohibits the orthogonality of the two phenyl groups of the biphenyl tetrazole ring system (Figure 1). Indeed, theoretical calculations have shown that the two phenyl rings are deviating by approximately 60^o^ from the co-planar position. These findings triggered our interest to study the conformational properties of other commercially available AT1R antagonists. Similar conformational features were observed for the AT1R antagonists eprosartan and telmisartan [25,26,27].

Among the AT1R commercial antagonists studied was valsartan. Interestingly, this molecule showed that due to the existence of an amide bond, it could exist in two distinct and almost isoenergetic *cis* and *trans* conformations.(1)
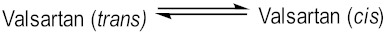


**Figure 2:**
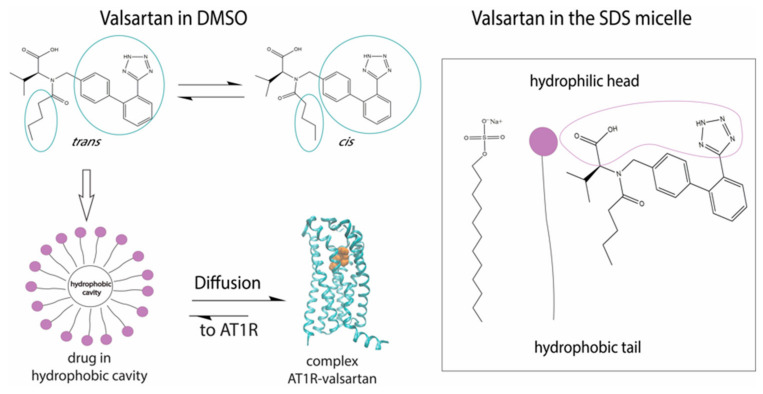
Mechanism of action of valsartan. The *trans* conformation of the drug is firstly encapsulated in the micelle and then diffused towards AT1R.

In DMSO solvent we discovered that the *trans:cis* ratio is 60:40%. Fangand his colleagues used ^1^H chemical shift analysis, proton relaxation rates and self-diffusion coefficient measurements in order to study the conformation of valsartan when embedded in sodium dodecyl sulfate (SDS) [28]. They observed that the *trans* conformation is favored versus the *cis* conformation (Figure 2). These results are in accordance with the results published by our research group, derived from high resolution solution NMR spectroscopy and in silico docking studies. In addition, the combination of NOE measurements and MD simulations showed that valsartan’s biphenyl rings and butyl chain exerted hydrophobic interactions with the alkyl group of SDS. Interestingly, their results revealed that the biological membrane plays an essential role in stabilizing the active state of valsartan. The understanding of the interactions between the sartan drugs and the membrane environment should facilitate the functional mechanism of these compounds with their receptor. The key role of the cellular membrane in sartans’ binding to transmembrane AT1 receptor indicates that the investigation of sartans should also involve AT1R:membrane interactions’ studies. They could also provide insight into the development of new approaches for drug discovery as these were performed by our groups and will be analyzed below.

Another computational investigation concerning the bioactive conformers of valsartan suggests that the molecule adopts its *cis* conformation in the receptor’s active site. This particular study aims to observe the changes of AT1R’s conformation when *cis* or *trans* valsartan enters the cavity. According to the MD simulations performed, *trans* conformation stabilizes the structure and locks AT1R in an inactive state, while the *cis* conformation causes the displacement of an a-helix of AT1R resulting in the expansion of the binding pocket. This extended binding pocket could serve as a potent binding point for the G-protein [29]. Similar helical displacement has been also observed by Khuraijam Dhanachandra Singh et al., when studying angiontensin’s II activation of AT1R. This molecular dynamics study suggests that a van der Waals interaction between Phe8 of angiotensin II and Ile288 of AT_1_R leads to a sequence of conformational changes in AT1R, resulting in the formation of G-protein binding point and the exposure of a receptor’s helix to cytoplasm [30].

The determination of the absolute conformation of the drugs targeting AT1R through 2D NOESY experiments has been also applied to 6-substituted aminocarbonylbenzimidazole derivatives [31] aiming to inhibit AT1R’s action.

**Figure 3:**
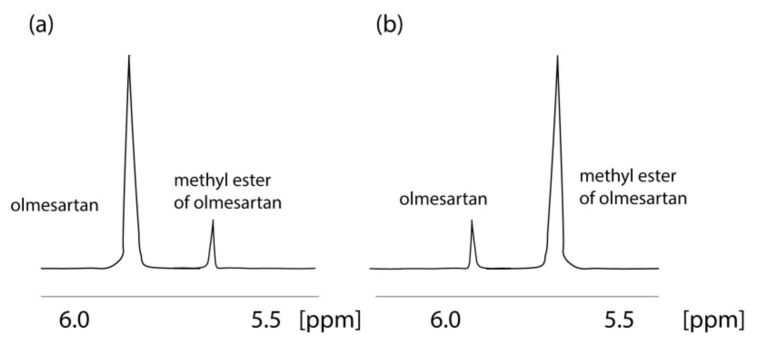
Formation of methyl ether of οlmesartan with time. ^1^H NMR spectra of 2 mg of olmesartan dissolved in CD_3_OD obtained on a Bruker Avance III spectrometer (Bruker Biospin GmbH, Reinsteten, Germany) operating at 600.11 MHz for ^1^H at 45 °C: (**a**) recorded 4 h after dissolution; (**b**) recorded after 52 months.

An important fact indicated by the application of NMR is that olmesartan is not stable in CD_3_OH solvent. The ^1^H NMR spectrum of olmesartan was altered due to its conversion to its methylated analog with time as confirmed also by mass spectrometry (MS) [32]. This observation led us to develop a simple synthesis for the methylated analog of olmesartan and to evaluate its biological activity. Interestingly, the methylated analogue was almost equipotent with olmesartan depicting that even a relevant biotransformation that could happen in the body, i.e., enzymatically via methyl transferases, will not significantly modify the bioactivity profile of olmesartan (Figure 3).

## 3. AT1R Antagonists: Membrane Bilayer Interactions

Three angiotensin II peptide antagonists ([Sar1]-AII(1-7)-NH2,[Sar1,Val5,Ala8]-AII and the AII antipeptide, [Glu1,Gly2,Val5,Val8]-AII) have been assessed by NMR and molecular docking studies in a lipid medium in order to determine the conformational requirements of the molecules for their AT1R binding. Under this study, it has been unveiled that histidine residue (6th residue) carries a pivotal role in the determination of the conformation of the peptide antagonists. The insightful NOEs obtained by these studies revealed the interactions between residues 1 and 6, when the antagonists were embedded in micelle solution. 1D and 2D ^1^H NMR spectra were obtained at different temperatures between 30 to 50 °C. Wilkes et al. discovered that the amide protons associated with residues 4 and 5 are more shielded from the aqueous environment than others participating in the peptide sequence [33].

As it has been already mentioned the exact mechanism of how the AT1R antagonists approach the receptor has not been unveiled and there are two potent scenarios concerning the drug binding mechanism of sartans to the AT1R. According to these theories, either sartans act directly on the AT1R or they exert their action through a two-step mechanism that involves their incorporation into the lipid bilayers and then their diffusion to the active site of the receptor.

**Figure 4:**
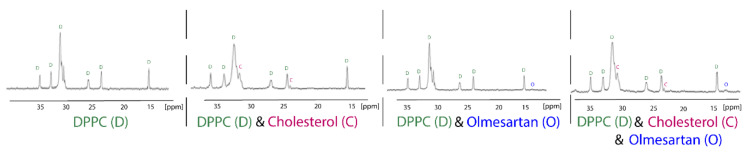
^13^C CP/MAS obtained at liquid crystalline phase of DPPC bilayers (45 °C) of the samples DPPC, DPPC/cholesterol (85:15), DPPC/olmesartan (80:20) and (DPPC/cholesterol)/olmesartan (80:20). ^13^C NMR-spectra were obtained at 150.80 MHz with a 600 MHz Varian spectrometer (Palo Alto, CA) and spinning rate 5 kHz. Sample preparation involved appropriate amounts of DPPC, olmesartan and cholesterol, diluted in chloroform/methanol mixed, dried under stream of argon and then stored under high vacuum overnight. Approximately 50 mg of the sample packed tightly into a zirconia rotor [35]. Reprinted from Biochimica et Biophysica Acta (BBA)—Biomembranes, 1808, Dimitrios Ntountaniotis, Gregor Mali, Simona Golic Grdadolnik, Halabalaki Maria, Alexios-Leandros Skaltsounis, Constantinos Potamitis, Eleni Siapi, Petros Chatzigeorgiou, Michael Rappolt, Thomas Mavromoustakos, Thermal, dynamic and structural properties of drug AT1 antagonist olmesartan in lipid bilayers, pages. 2995–3006, Copyright (2011), with permission from Elsevier.

^13^C Cross-polarization and magic-angle spinning (CP/MAS) is a tremendously useful technique used to explore drug:membrane interactions, since the putative incorporation of the molecule into the membrane could be detected by observing the drug’s peaks in the spectra. Chemical shift’s changes and broadening of drug’s peaks can reveal very useful information about the dynamic properties of the molecules into the lipid bilayers. Anisotropic interactions between nuclei, which are usually averaged by Brownian motion in liquid samples, cause significant line broadening in solid state NMR, but they can be averaged to zero by spinning the sample very rapidly at the magic angle (54.74° with respect to the direction of the magnetic field). Cross-polarization (CP) is the most important signal enhancement technique in solid-state NMR. Polarization is first transferred from the abundant spins typically ^1^H, to the dilute spins typically ^13^C and the signal from the dilute spins C is then observed. The following ^13^CP/MAS spectra of the hydrophobic region of dipalmitoylphospatidylcholine (DPPC), DPPC/cholesterol, DPPC/olmesartan and DPPC/cholesterol/olmesartan spectra shows the direct incorporation of cholesterol, since new peaks arise due to its presence. The direct presence of olmesartan in the bilayers is also detected due to the changes it causes to the chemical shifts of DPPC bilayers in this hydrophobic region.It should be notated that olmesartan does not influence the DPPC/cholesterol chemical shifts (Figure 4). This is an intriguing effect as previous studies have shown that the AT1R antagonist losartan does not prefer lipid rafts containing cholesterol. It is evident that AT1R antagonists, in case they act through the mechanism involving lipid bilayers, they do not tend to approach the cholesterol regions of the membrane [34,35].

Hydrated undecoupled ^31^P NMR spectra of the non-oriented and unsonicated hydrated phospholipid bilayers indicates one broad line that covers approximately a 50 ppm line width. The line shape of the observed powder-type spectrum is attributed to chemical shift anisotropy and proton-phosphorus dipolar interactions [36,37]. The ^31^P-^1^H dipolar broadening is removed by ^1^H decoupling, either in a single free induction decay experiment or in a cross-polarization experiment invented by Hartmann and Hahn in 1962 [38]. ^31^P is one of the most useful nuclei as it exhibits 6.6% sensitivity relatively to ^1^H. ^31^P NMR is a very powerful tool widely applied in model and biological membranes [39].

**Figure 5:**
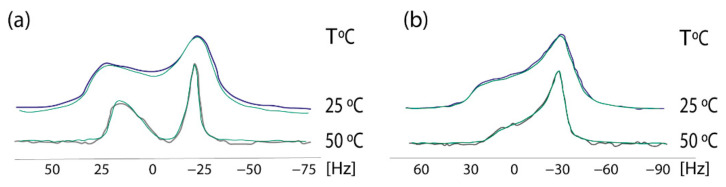
Experimental (black and purple lines) and simulated (green lines) of 31P NMR Scheme 0. (**a**) DPPC alome (**b**) DPPC with candesartan cilexetil at the temperatures of 25 and 50 °C. Solid state ^31^P CP NMR. Spectra were obtained on a Bruker MSL-400 NMR spectrometer (Rheinstetten, Germany) operating at 161.977 MHz using high-power ^1^H-decoupling. Each spectrum was an accumulation of 1000 scans. The standard CP pulse sequence of the Bruker library was used with the following acquisition parameters: recycling delay 4 s, contact time 5 ms, acquisition time 1 ms, and π/2 pulse for proton 7 ms. The contact time was chosen to give optimal spectra after testing at 1, 3, and 5 ms. The sample was revolved in a 4-mM rotor at a low frequency of 25 Hz. ^31^P resonance was referenced to H_3_PO4 (85% in D_2_O). All simulations were obtained imposing Lorentzian spin packets and automated fitting using the downhill simplex algorithm with a convergence criterion of 0.01 [43]. Reprinted from Biochimica et Biophysica Acta (BBA)—Biomembranes, 1818, Charalambos Fotakis, Grigorios Megariotis, Dionysios Christodouleas, Eftichia Kritsi, Panagiotis Zoumpoulakis, Dimitrios Ntountaniotis, Maria Zervou, Constantinos Potamitis, Aden Hodzic, Georg Pabst, Michael Rappolt, Gregor Mali, Johanna Baldus, Clemens Glaubitz, Manthos G. Papadopoulos, Antreas Afantitis, Georgia Melagraki, Thomas Mavromoustakos, Comparative study of the AT1 receptor prodrug antagonist candesartan cilexetil with other sartans on the interactions with membrane bilayers, pages. 3107–3120 Copyright (2012), with permission from Elsevier.

In 2009 we established a cross-polarization (CP) ^31^P NMR broadline simulation methodology for studying the effects of drugs in phospholipid bilayers. Based on seven-parameter fittings, this methodology provided information concerning the conformational changes and dynamic effects of drugs in the polar regions of phospholipid bilayers [40]. A simplified dynamical model was used, with the studied dipalmitoylphosphatidyl choline (DPPC) multilamellar bilayers that were considered to be immobilized in the time-scale of the NMR experiment, whereas the lipid molecules were assumed to perform fast overall rotational diffusion in both the liquid crystalline and in the more organized gel phase. A detailed theory of the broadline CP ^31^P NMR simulations of fully hydrated DPPC dispersions in the form of lipid bilayers has been published earlier [41]. ^13^CP/MAS experimental indicated that AT1R antagonists indeed affect the DPPC head-group. In order to get a deeper understanding, ^31^P static NMR spectroscopy was applied. The establishment of this methodology was achieved using losartan. In a subsequent study the prodrug of candesartan, candesartan cilexitil, has been also used as an example. These spectra pinpointed that the prodrug candesartan cilexitil modifies the isotropic shift towards lower values and abolishes the deep minimum observed in the ^31^P broadline spectra in the gel phase (Figure 5). More detailed information of the techniques and the plethora of information can be found in our previous research work [42].

Solid state ^2^H NMR is also a valuable tool for getting information on the dynamic properties of a drug embedded in the membrane bilayers. The experiment utilizes the quadrupole echo pulse sequence ((π/2)x − τ − (π/2)y). Thus, the pulse sequence consists of a pair of 90°-phase-shifted π/2 pulses separated by time τ. Due to the interaction between the electric quadrupole moment and the electric field gradient at the nuclear site, the deuterium nucleus gives rise to a doublet. The anisotropic nature of this interaction gives two spectral lines having a frequency difference of ν1 − ν2 = (3/4)Α_Q_ (3cos^2^θ − 1), which depends on the angle θ between the magnetic field and the axis of molecular ordering. In the aqueous phospholipid bilayers in the liquid crystalline phase all values of θ are possible and the spectrum consists of two principal peaks that correspond to θ = 90° (perpendicular edges) and two shoulders to θ = 0° (parallel edges). The separation between the two 90°-edges is defined as the quadrupolar splitting (Δν_Q_). At temperatures below the main phase transition, the axial diffusion rate is slow or intermediate and appears a conical or rounded spectrum which does not show sharp features. The study of the spectral shape as a function of temperature and the changes in ΔvQ because of the drug incorporation, delivers information on the dynamic properties of the lipid bilayers in the specific labeled region.

**Figure 6:**
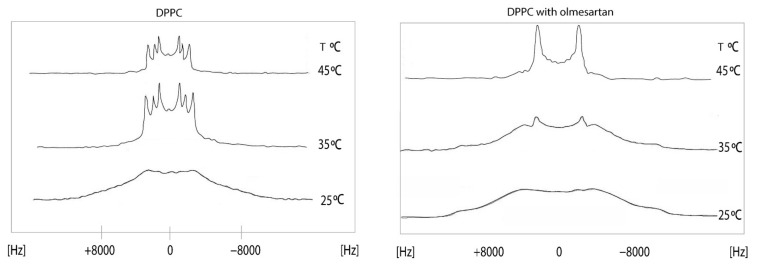
^2^Η NMR spectra of 1,2[2,2-^2^H]-DPPC bilayer (**left**) at a temperature range of 15–45 °C and 1,2 [2,2-^2^H]-DPPC bilayer with olmesartan (20% molar ratio) was added (**right**). ^2^H solid-state NMR spectra were recorded on a BrukerAvance 600 MHz WB spectrometer equipped with a 4 mm HX double resonance MAS probe. Spectra were recorded without spinning the 4 mm MAS rotor using a solid echo sequence with an echo delay of 35 μs and a pulse length of 2.5 μs [45]. Reprinted from Biochimica et Biophysica Acta (BBA)—Biomembranes, 1838, Dimitrios Ntountaniotis, Tahsin Kellici, Andreas Tzakos, Pinelopi Kolokotroni, Theodore Tselios, Johanna Becker-Baldus, Clemens Glaubitz, Sonyan Lin, Alexandros Makriyannis, Thomas Mavromoustakos, The application of solid-state NMR spectroscopy to study candesartan cilexetil (TCV-116) membrane interactions. Comparative study with the AT1R antagonist drug olmesartan, pages. 2439–2450, Copyright (2014), with permission from Elsevier.

The effects of olmesartan in the DPPC bilayers were studied using solid state stationary ^2^H NMR spectroscopy. It can be observed that in the gel phase, up to 30 °C, the spectrum is featureless. Above this temperature, the dynamics of the lipid bilayers allows the appearance of quadrupolar splittings but still not quite evident to measure. This is indicative that DPPC bilayers are in the ripple phase. In the liquid crystalline phase (45 °C), the spectrum of DPPC bilayers shows three quadrupolar splittings 11,9 (2-S), 17,2 (2-R) and 26,2 (-CD2) kHz [44]. The presence of olmesartan causes a significant increase of ΔνQ at 2′-S and a decrease at 2′-R Thus, this position has a bimodal effect. The spectra in the temperature range of 15–45 °C (Figure 6) pinpoints that between 15 and 30 °C the preparation containing olmesartan is in the gel phase and between 35 and 45 °C it is altered to the liquid crystalline phase. The presence of olmesartan abolishes the pre-transition state.

Similar spectra can be also obtained for other labeling schemes at different positions and thus this could reveal the selectivity of the drug to participate in specific topographic position in DPPC bilayers. In addition, comparative studies with other prodrugs or AT1R antagonists can be established using specific deuteration [45].

Another aspect that should be investigated in order to detect the preference of sartans for lipid bilayers should be the ionization states and the pKa of each sartan. A Quantum Mechanics (QM) study on ionization of three sartans (losartan, irbesartan and valsartan) in aqueous media and on interactions with surfactant micelles has been performed by Popović-Nikolić et al. This study suggested that sartans in their amphiprotic form interact predominantly with michelle surfaces, while presenting quite high affinity with the lipid area. Similar results have been acquired for their diprotic acid’s form, but under this protonation state sartans do not interact stongly with the micelle [46].

## 4. Formulation of AT1R Antagonists with Cyclodextrins

Angiontensin II could be also used as a prototype for the evaluation of the inclusion complexes of drugs in cyclodextrins. As observed by Lula et al. through rigorous 2D TOCSY, 2D HSQC and 2D HMBC NMR studies, angiontensin II can be encapsulated in a cyclodextrin molecule and its three-dimensional conformation could be established. The study concludes that among all angiontensin II residues, tyrosine is the most favoured for the formation of the inclusion complex. These results have been further analyzed by quantum mechanics/molecular mechanics (QM/MM) simulations [47].

Furthermore, many AT1R antagonists have been encapsulated in cyclodextrin-carriers because of their high lipophilicity and low bioavailabilty and have been studied for their stability and action, [48]. The complex of valsartan engulfed in cyclodextrin has been thoroughly characterized by 2D ROESY NMR, differential scanning calorimetry (DSC), and other biophysical methods in order to detect the most efficient method of complex preparation. 2D ROESY NMR experiments conducted by Carlos Eduardo de Matos Jensen et al.indicated that the “cyclodextrin-sartan” complex prepared with freeze-drying method provided the best results concerning its stability, bioavailability and anti-hypertensive evaluation [49]. Losartan-cyclodextrin complex has been also extensively studied [50] and presented very promising results. In particular, according to De Paula et al., a new long-lasting oral preformulation of losartan complexed in cyclodextrin has been discovered and characterized by 2D ROESY NMR experiments. This complex prolongs the time of losartan’s action, increases its bioavailabilty and achieves a quick onset of action [51].

**Figure 7:**
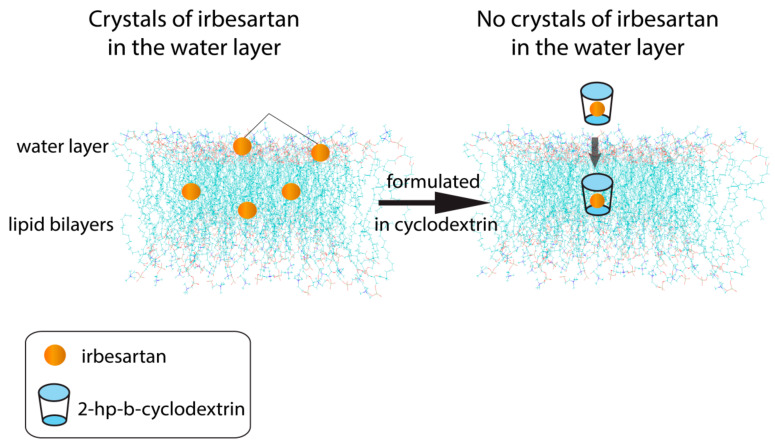
Irbesartan molecules crystallized into the lipid bilayers, although no crystals are observed when the drug is in complexation with 2-hydroxy-propyl-cyclodextrin.

Irbesartan is another beneficial sartan either alone or in combination with other drugs acting against hypertension, cancer, and diabetes, so it can be characterized as a multi-dynamic drug [52,53,54,55,56,57,58,59]. In a previous study we investigated the interactions of the polydynamic drug irbesartan (IRB) in phospholipid bilayers utilizing an array of biophysical techniques ranging from (DSC), small angle X-ray scattering (SAXS), electrospray ionisation mass spectrometry (ESI-MS), and solid-state nuclear magnetic resonance (ssNMR) spectroscopy. IRB was incorporated in the lipid membrane core and affected the phase transition properties of the DOPC bilayers. SAXS studies revealed that IRB alone does not display perfect solvation, as some coexisting IRB crystallites are present. However, in its complexed form, IRB gets fully solvated in the membranes (Figure 7) [60] showing that IRB encapsulation in hydroxyl-propyl-b-cyclodextrin (HP-β-CD) (Figure S1) may have beneficial effects in its absorption distribution metabolism and excretion (ADME) properties [61].

In vitro pharmacological results verified that this inclusion complex not only preserves the binding affinity of IRB for the AT1R receptor, but it also slightly enhances it. As the complex formulation lacks the problems of the tablet, our approach is a promising new way to improve the efficiency of IRB in the organism [50,62,63,64,65,66,67,68].

## 5. Formulation of AT1R Antagonists with Copolymer Micelles

**Figure 8:**
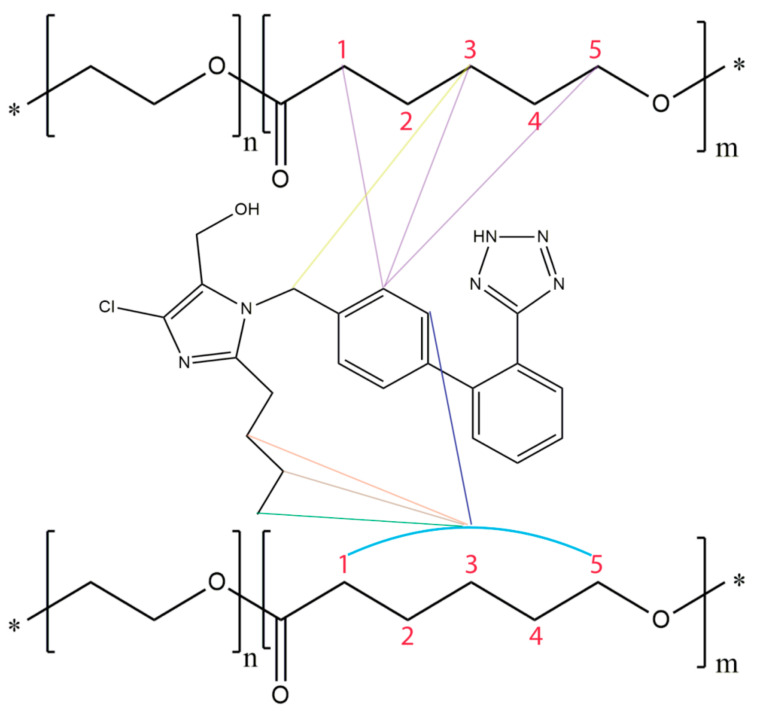
Eminent hydrophobic interactions when losartan approaches PEO-b-PCL micelles. The colorful lines represent the NOEs between losartan and the polymer. The “*” sign denotes the presence of end groups in the polymer chain.

Biocompatible amphiphilic copolymers have been used as drug nanocarriers as they can enhance their solubility, prolong circulation times and control drug release [1,2,3,4,5,6,7,8,9]. Specifically, block copolymer micelles contain a hydrophobic core that can engulf hydrophobic molecules such as sartans. Such drug encapsulation will result in the above mentioned health benefit results [69,70,71,72,73,74,75,76,77,78,79,80,81,82]. Poly(ethylene oxide)-b-poly(ε-caprolactone) (PEO-b-PCL) micelles have been synthesized by the Pispas’s group and losartan has been loaded as a guest to these nanocarriers [55]. 2D NOESY experiments confirmed losartan’s successful encapsulation and provided direct evidence of the interactions of the biphenyl ring and butyl chain of losartan with the methylene groups of PCL [64,65] (Figure 8).

## 6. Conclusions

This review provides an array of examples where NMR spectroscopy has provided valuable information aiding towards the rational drug design process against hypertension. Although hypertension has been a well-known health risk factor for years and a considerable experience has been accumulated with the successful development of the new generation of sartans, there is still critical information that remains elusive. For instance, the accurate mechanism that sartans approach their membrane bound GPCR AT1R is still elusive. This question could be unveiled through exploring the interaction of sartans with model membranes, since the cell membrane plays a key-role in their binding mechanism to the receptor site. Furthermore, in order to develop sartans’ formulations and ameliorate their bioavailability and their pharmacodynamic profile, an atomic level analysis of their interactions with the relevant formulation compounds (molecules-carriers) should also be conducted.

An important constituent of the rational drug design process is the knowledge of putative bioactive conformation of the drug. This knowledge is of paramount importance especially when the drug acts on the receptor site, since its bioactive conformation is responsible for regulating its biological action. Along these lines, NMR is a powerful tool that could allow charting not only the conformational population that the drug has in different solvents, but also it could enable recording information on the potent bioactive conformation of the drugs. This could be achieved through the multitude information that can be generated using NMR spectra not only both on different states (solid or liquid) of the drug but also in the presence of its formulation’s agents like cyclodextrins and model lipid bilayers.

As it has been already mentioned, AT1R antagonists, and generally the majority of the drugs, act on transmembrane receptors such as GPCRs. In such cases, the drug can exert its action directly to the receptor or indirectly through its diffusion mechanism in the lipid bilayers [83]. Solid-state NMR spectroscopy is a powerful technique for studying drug:membrane interactions, and it could be quite useful in unveiling the mechanism of AT1R antagonists’ action with the lipid bilayers. Specific deuteration of drugs or phospholipids is used for providing information at atomic level, when a drug acts on the membrane bilayers. Specifically, ^2^H stationary solid state NMR spectroscopy is an indispensable tool for providing this information. This knowledge could open novel directions for rational drug design against hypertension.

Studies on the drug:receptor or drug:DNA interactions not covered in this review complement the applications of NMR spectroscopy on rational drug design and show its expanded applications.

In summary, NMR is a technique that is indispensable at all stages in the drug discovery pipeline [84] and development and especially in constructing more effective compounds for the treatment of hypertension [85]. The use of NMR spectroscopy using the well established experiments such as saturation transfer difference (STD), water-ligand observed via gradient spectroscopy (water LODGY [85]), transfer NOE (trNOE) and structure activity relations (SAR) by NMR [86,87,88,89] will expand the scope of the rational drug design. These experiments are complementary to X-ray crystallography. Additional biophysical studies of drug:AT1 and drug:lipid bilayer interactions must be also applied in the field. For example, fluorescence studies, measurements of lateral diffusion rates, scanning optical microscopy to identify membrane microdomains, atomic force microscopy to study interacting forces in phospholipid bilayers containing AT1 antagonists. It is quite surprising that there are only limited biophysical techniques reported, i.e., electron spin resonance (ESR) studies and differential scanning calorimetry (DSC) studies [90,91].

## Supplementary Materials

The following are available online, Figure S1: 1H NMR spectrum of DOPC loaded with “cyclodextrin-irbesartan” complex.
