# Supplementary material for: On the Rational Drug Design for Hypertension through NMR Spectroscopy
Source: Molecules. 2020 Dec 22;26(1):12. doi: 10.3390/molecules26010012 (PMC7792925; doi:10.3390/molecules26010012)
Supplement: Supplementary file 1 [file molecules-26-00012-s001.pdf]

Supplementary Material

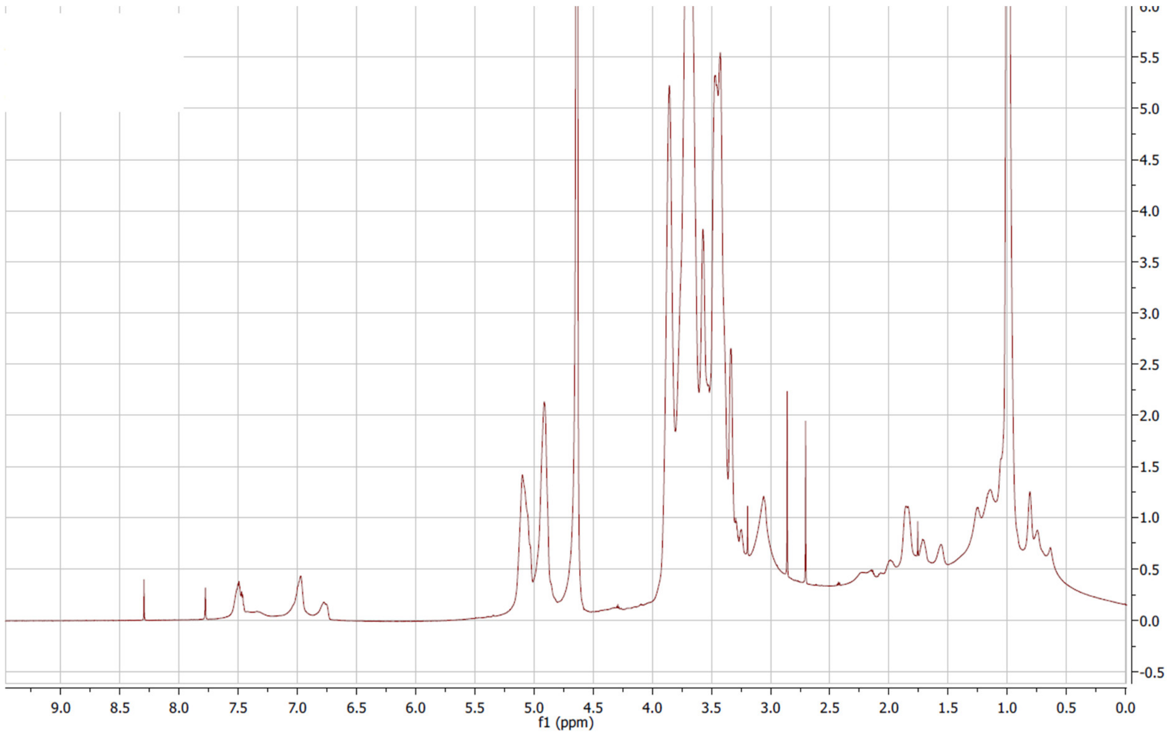

Figure S1. ^{1}H NMR spectrum of DOPC loaded with "cyclodextrin-irbesartan" complex. In the spectrum provided below, we observe that when irbsesartan is complexed with cyclodextrin and then is embedded in lipid bilayes, it gets fully solvated. In particular, ^{1}H NMR spectrum demonstrates the peaks of irbesartan resonating in the aromatic region (6.8–8.4 ppm). This is indicative that the drug is released from cylodextrin and is embedded in the DOPC bilayer core.
